# Tobacco Smoke and Electronic Cigarette Vapor Alter Enhancer RNA Expression That Can Regulate the Pathogenesis of Lung Squamous Cell Carcinoma

**DOI:** 10.3390/cancers13164225

**Published:** 2021-08-23

**Authors:** Joseph C. Tsai, Omar A. Saad, Shruti Magesh, Jingyue Xu, Abby C. Lee, Wei Tse Li, Jaideep Chakladar, Mark M. Fuster, Eric Y. Chang, Jessica Wang-Rodriguez, Weg M. Ongkeko

**Affiliations:** 1 Department of Surgery, Division of Otolaryngology—Head and Neck Surgery, UC San Diego School of Medicine, San Diego, CA 92093, USA; jctsai@ucsd.edu (J.C.T.); omar_saad@ucsb.edu (O.A.S.); smagesh@ucsd.edu (S.M.); j1xu@ucsd.edu (J.X.); acl008@ucsd.edu (A.C.L.); wtl008@ucsd.edu (W.T.L.); jchaklad@ucsd.edu (J.C.); 2 Research Service, VA San Diego Healthcare System, San Diego, CA 92161, USA; 3 VA San Diego Healthcare System, Medical and Research Sections, La Jolla, San Diego, CA 92161, USA; mfuster@health.ucsd.edu; 4 Department of Medicine, Division of Pulmonary and Critical Care, University of California, La Jolla, San Diego, CA 92037, USA; 5 Department of Radiology, University of California, San Diego, CA 92093, USA; e8chang@health.ucsd.edu; 6 Radiology Service, VA San Diego Healthcare System, San Diego, CA 92161, USA; 7 Department of Pathology, UC San Diego School of Medicine, San Diego, CA 92093, USA; jessica.wang-rodriguez@va.gov; 8 Pathology Service, VA San Diego Healthcare System, San Diego, CA 92161, USA

**Keywords:** LUSC, eRNA, tobacco smoke, electronic cigarette smoke, vaping

## Abstract

**Simple Summary:**

It is well established that tobacco smoke is the key player in lung squamous cell carcinoma (LUSC) pathogenesis, and there is growing evidence that electronic cigarette (e-cigarette) vapor may also cause LUSC. Recently, several studies have associated tobacco smoke with differential enhancer RNA (eRNA) expression. However, the effects of tobacco smoke and e-cigarette vapor on eRNA expression in correlation to LUSC outcomes have not been fully elucidated. This study demonstrates that tobacco smoke and e-cigarette vapor may decrease DNA methylation and increase chromosomal alterations at key sites, which ultimately upregulate the expression of oncogenic eRNAs and downregulate the expression of tumor-suppressing eRNAs. Subsequently, we demonstrate that these eRNAs may have altered interactions with immune cells to promote LUSC pathogenesis and reduced patient survival. We hope our results can be validated in future studies, and the key eRNAs we identified may be used as effective targets for more specialized treatments for smoking-mediated LUSC.

**Abstract:**

Tobacco is the primary etiologic agent in worsened lung squamous cell carcinoma (LUSC) outcomes. Meanwhile, it has been shown that etiologic agents alter enhancer RNAs (eRNAs) expression. Therefore, we aimed to identify the effects of tobacco and electronic cigarette (e-cigarette) use on eRNA expression in relation to LUSC outcomes. We extracted eRNA counts from RNA-sequencing data of tumor/adjacent normal tissue and before/after e-cigarette tissue from The Cancer Genome Atlas (TCGA) and the Gene Expression Omnibus (GEO), respectively. Tobacco-mediated LUSC eRNAs were correlated to patient survival, clinical variables, and immune-associated elements. eRNA expression was also correlated to mutation rates through the Repeated Evaluation of Variables Conditional Entropy and Redundance (REVEALER) algorithm and methylated sites through methylationArrayAnalysis. Differential expression analysis was then completed for the e-cigarette data to compare with key tobacco-mediated eRNAs. We identified 684 downregulated eRNAs and 819 upregulated eRNAs associated with tobacco-mediated LUSC, specifically, with the cancer pathological stage. We also observed a decrease in immune cell abundance in tobacco-mediated LUSC. Yet, we found an increased association of eRNA expression with immune cell abundance in tobacco-mediated LUSC. We identified 16 key eRNAs with significant correlations to 8 clinical variables, implicating these eRNAs in LUSC malignancy. Furthermore, we observed that these 16 eRNAs were highly associated with chromosomal alterations and reduced CpG site methylation. Finally, we observed large eRNA expression upregulation with e-cigarette use, which corresponded to the upregulation of the 16 key eRNAs. Our findings provide a novel mechanism by which tobacco and e-cigarette smoke influences eRNA interactions to promote LUSC pathogenesis and provide insight regarding disease progression at a molecular level.

## 1. Introduction

Lung cancer is the leading cause of cancer-related deaths in men and women in the United States, accounting for 25% of all cancer-related deaths [1]. Lung cancer can be divided into small cell lung cancer and non-small cell lung cancer, with non-small cell lung cancer accounting for 80% of all types of lung cancer. Non-small cell lung cancer has two primary histological classifications: lung squamous cell carcinoma (LUSC) and lung adenocarcinoma (LUAD). LUSC affects over 100,000 [2,3] people annually and is one of the most aggressive cancers, with a 10% 5-year survival rate. LUSC pathogenesis is largely influenced by several risk factors, including tobacco use and alcohol consumption. As such, it is considered a very heterogeneous cancer [4,5]. The unpredictable nature and poor survival rates associated with LUSC highlight the importance of closely examining etiologies and other risk factors that may influence tumor progression.

Enhancer RNAs (eRNAs) are non-coding RNAs that are transcribed from enhancer regions of DNA [6]. eRNAs play critical roles in a wide range of functions, including regulating gene expression, modulating acetylation and methylation, and regulating other transcriptional factors. Previous studies have suggested that eRNAs may be clinically significant biomarkers in specific cancers [7]. In fact, the immune-related eRNA AP001056.1 is associated with negative survival outcomes in LUSC patients [8]. Similarly, eRNA TBX5-AS1 was found to be correlated with survival outcomes in LUSC [9]. Furthermore, studies have found that there were 337 eRNAs associated with smoking in LUSC, indicating that smoking may alter eRNA expression patterns [7]. Together, these recent studies suggest that eRNAs play a critical role in LUSC prognoses and that tobacco smoke may affect eRNA interactions, driving LUSC pathogenesis.

Previous studies indicate that 80% of LUSC patients were former smokers [10], and tobacco smoke has been identified as the primary etiological agent of LUSC. It has been widely established that tobacco smoke contains a multitude of compounds, including 60 carcinogens such as alkylated PAHs and N-nitrosamines [11,12]. These constituents covalently bind to DNA, forming DNA adducts. If unrepaired, these DNA adducts may lead to errors during DNA replication, resulting in the accumulation of permanent somatic mutations. These somatic mutations may then promote the activation of oncogenes and the inactivation of tumor-suppressor genes, driving tumor initiation and progression. This has been observed in numerous LUSC studies. Previous studies have found that tobacco smoke increases the somatic mutation load in LUSC, resulting in genetic damage that can increase susceptibility to cancer [13]. Tobacco smoke also results in genetic alterations, such as in the tumor-suppressor gene TP53, which can promote tumor development [14]. Additional studies suggest that tobacco smoke leads to increased infiltration of T follicular helper cells and the activation of resting CD4+ memory T cells [15]. These immune cells may interact with tissue-specific differentially expressed long non-coding RNAs (DElncRNAs), such as PWRN1 and APOBEC1, increasing tumor proliferation. Lastly, tobacco smoke has been found to correspond to the development of airway lesions in LUSC [6,13]. However, although an abundance of evidence suggests that tobacco smoke is implicated in LUSC, many of the molecular and genetic mechanisms for tobacco-smoke-mediated LUSC pathogenesis have not been fully elucidated [6,13,14,15]. Given that eRNAs may have clinical utility in LUSC prognoses and have been found to be dysregulated in tobacco-smoke-onset LUSC, we believe that eRNA dysregulation may have a role in a possible molecular mechanism for tobacco-smoke-mediated LUSC.

In addition to tobacco smoke, previous studies have suggested that electronic cigarette (e-cigarette) smoke also plays a role in inducing lung cancer and driving advanced tumor phenotypes [16]. It is well established that cancer is a genetic disease associated with DNA alterations and mutations [17]. Accordingly, e-cigarettes contain various substituents, such as carbonyl compounds [18], acrolein [19], and nicotine substances [18], which are highly capable of causing DNA breakages, inhibiting DNA repair functions, and releasing reactive oxygen species [18]. As such, these substituents increase the risk for lung cancer and promote tumor growth. Moreover, e-cigarette aerosol is very small, allowing it to propagate deeply in lung tissue, resulting in more extensive DNA damage and adduct formation [16]. Therefore, there is strong evidence implicating e-cigarette smoke in lung cancer metastasis and induction. However, similar to tobacco-smoke-mediated LUSC, the exact mechanisms by which e-cigarette smoke may influence intratumor interactions to promote lung cancer pathogenesis remain unclear.

In this study, we aim to analyze the effects of tobacco smoke and e-cigarette smoke on eRNA expression in LUSC. Next, we aim to investigate novel mechanisms by which tobacco smoke may alter the expression of particular eRNAs. We will then characterize the effect of differential eRNA expression on immune cell abundance, patient survival, and clinical variables in order to further elucidate the implications of these eRNAs in LUSC pathogenesis and malignancy. Using RNA sequencing data for 18 nonsmoking patients and 129 smoking patients from The Cancer Genome Atlas (TCGA), we performed differential expression analysis to identify eRNA molecules that were significantly dysregulated and correlated to tobacco use. Through survival analysis and clinical variable correlations, we aimed to associate significantly dysregulated eRNAs to patient survival and several clinical variables, including cancer pathological stage and tumor necrosis percent. In addition, we examined correlations between eRNA expression and immune cell infiltration to understand how eRNAs might alter immune cell abundance. We then assessed the effects of copy number variants and mutations on eRNA expression through the Repeated Evaluation of VariablEs conditionAL Entropy and Redundancy (REVEALER) algorithm to identify potential mechanisms by which tobacco smoke might alter eRNA expression. We also examined correlations between significantly dysregulated eRNAs and methylated sites through methylationArrayAnalysis from Bioconductor. Finally, we investigated the impact of e-cigarette smoke on eRNA expression to evaluate how e-cigarette smoke may modulate eRNA expression to alter LUSC outcomes.

## 2. Materials and Methods

### 2.1. Data Acquisition

Raw whole transcriptome RNA sequencing data for tumor and adjacent normal tissue were downloaded from the TCGA legacy archive for 49 LUSC adjacent normal samples, 134 tobacco-smoke-onset LUSC samples, and 18 nonsmoking-onset LUSC samples [20]. Patient clinical data were obtained from Broad Genome Data Analysis Center (GDAC) Firehose [21].

### 2.2. Extraction of eRNA Counts

eRNA counts were processed from RNA sequencing data through direct alignment to an eRNA sequence reference file using the Bedtools framework for genomic analysis [22]. The number of segments in the samples found to be overlapping with the reference file was extracted as eRNA read counts for further analyses. Five tobacco smoke samples failed to yield successful alignments and were eliminated from the tobacco-smoke-onset LUSC cohort, resulting in a final dataset of 129 samples.

### 2.3. eRNA Differential Expression Analysis between Cohorts

LUSC patients were separated into distinct cohorts based on their smoking history to limit the effect of confounding variables. The two cohorts included lifelong nonsmoking LUSC patients and tobacco smoking LUSC patients. Differentially expressed eRNAs were analyzed separately in the two cohorts in comparison to adjacent normal LUSC samples using the edgeR library. Significantly differentially expressed eRNAs were identified and filtered based on fold change and *p*-value (log fold change (FC) > 1 and *p*-value < 0.05).

### 2.4. Correlation of eRNA Expression to Clinical Variables and Patient Survival

Significantly dysregulated eRNAs from differential expression analysis in both the tobacco-smoke-onset LUSC cohort and nonsmoking LUSC cohort were further assessed for correlations to clinical variables. Using the Kruskal–Wallis test in the edgeR library, we correlated eRNA expression to 6 clinical variables from The Cancer Genome Atlas (TCGA): cancer status, cancer pathological stage, cancer pathological m stage, cancer pathological n stage, cancer pathological t stage, and cancer vital status. Spearman correlation was then performed to compare eRNA expression to tumor necrosis percent and percent stromal cell expression. The Spearman test had to be completed for these two variables as they are continuous, not discrete variables.

eRNA expression was also correlated to patient survival through Survival Analysis in the edgeR library, and results were plotted using the Cox proportional hazards regression. eRNA read counts were used as the primary binary variable, with eRNA expression designated as above or below the median expression value. The time to death and status of each patient were used as measures of patient survival, and relevant clinical data were obtained from the Broad GDAC Firehose. Significant correlations were filtered by *p*-value (*p*-value < 0.05).

The total amount of eRNAs significantly correlated to each of the variables across all patients was compared between the nonsmoking and smoking LUSC cohorts in bar plots.

### 2.5. Correlation of eRNA Expression to Immune Associated Elements

The Tumor Immune Estimation Resource (TIMER) software was used to profile immune cell abundance in both tobacco-smoke-onset LUSC and nonsmoking-onset LUSC samples [23]. The TIMER software required an input matrix of gene expression read counts from both respective LUSC cohorts and provided a quantitative measure of immune cell infiltration in the patient samples, including dendritic cells, macrophages, CD8 T-cells, CD4 T cells, B cells, and neutrophils. Significant correlations were plotted using the Spearman test and filtered based on *p*-value (*p*-value < 0.05).

A bar plot of the immune abundances of patients in the nonsmoking and smoking LUSC cohorts was made with standard deviation error bars and asterisks indicating the resulting *p*-value of homoscedastic *t*-test between the two cohorts. The total amount of eRNAs significantly correlated to each of the immune cells across all patients was compared between the two cohorts in another bar plot.

### 2.6. Correlation of eRNA Expression to Copy Number Variants and Mutations

The annotation files for mutations and copy number variations (CNVs) were compiled into a binary input file for the program Repeated Evaluation Of Variables Conditional Entropy And Redundancy (REVEALER) [24], designed to computationally identify a set of specific CNVs and mutations most likely responsible for the change in the activity of a target profile. The target profile was defined in our study to be the expression of our panel of 16 key eRNAs. In order to identify a set of the most relevant genomic alterations, REVEALER runs multiple iterations of the correlation algorithm. The genomic feature exhibiting the strongest correlation in each run serves as a seed for the successive run. A seed is a particular mutation or copy number gain or loss event that most likely accounts for the target activity. When given a seed, REVEALER will focus correlations on patients with altered target activity not accounted for by the seed. Since we do not know which genomic alteration is responsible for the dysregulation of each eRNA, we designated the seed for the first iteration to null. The maximum number of iterations was set to three, and significant correlations were filtered by *p*-value and conditional information coefficient (CIC) (*p*-value < 0.05 and CIC >|0.3|).

### 2.7. Correlation of eRNA Expression to Methylated CpG Sites

DNA methylation 450k sequencing data were downloaded from the Genomic Data Commons (GDC) data portal for the tobacco-smoke-onset LUSC samples, nonsmoking-onset LUSC samples, and adjacent normal samples. Using a modified workflow of methylationArrayAnalysis from Bioconductor [25], we converted B-values to M-values and then performed probe-wise differential methylation analysis on the matrix of M-values in limma to obtain t-statistics and *p*-values for each CpG site [26]. The Kruskal–Wallis test was then used to correlate expression of the panel of 16 key eRNAs to the extent of methylation at each site.

### 2.8. Electronic Cigarette Smoke Dataset Acquisition and Differential Expression Analysis

RNA-sequencing data of small airway epithelium (SAE, New York, NY, USA) and alveolar macrophages (AM, New York, NY, USA) were obtained from the National Center for Biotechnology Information (NCBI) Gene Expression Omnibus (GEO) under accession code GSE85121. In this study, SAE and AM of 10 healthy lifelong nonsmokers were sampled prior to electronic cigarette use. Following acute electronic cigarette inhalation, SAE and AM in the same subjects were sampled once more [27].

We employed the same methods as with the TCGA LUSC samples to extract eRNA read counts and identify significantly differentially expressed eRNAs in the electronic cigarette smoke samples. eRNA read counts were extracted using the Bedtools framework for genomic analysis, and differential expression analysis was conducted using the edgeR library. Significantly dysregulated eRNAs were filtered by *p*-value and log fold change (log fold change (FC) > 1 and *p*-value < 0.05). We analyzed SAE and AM samples in separate cohorts to limit the effects of confounding variables. eRNA expression in the SAE and AM samples prior to electronic cigarette smoke inhalation was compared to eRNA expression in SAE and AM samples following acute electronic cigarette smoke inhalation.

## 3. Results

### 3.1. Identification of Smoking-Dysregulated eRNAs

**Figure 1:**
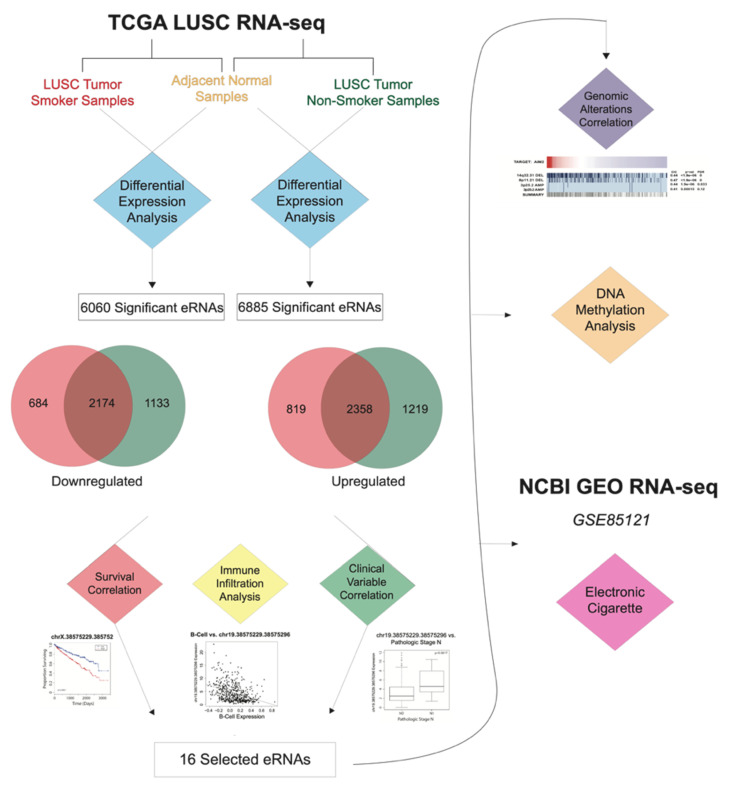
Project overview. Schematic of project workflow and analyses. Datasets were acquired from the Cancer Genome Atlas (TCGA) and the National Center for Biotechnology Information (NCBI) Gene Expression Omnibus (GEO). Tobacco-smoke-onset LUSC, nonsmoking-onset LUSC, and electronic cigarette smoke lung samples were analyzed in separate cohorts to limit any confounding variables. Differentially expressed eRNAs were filtered by log fold change and false discovery rate (log fold change (FC) > 1 and false discovery rate (FDR) < 0.05), and significantly dysregulated eRNAs were identified and used in survival analysis, immune infiltration analysis, and clinical variable correlations. A total of 16 key eRNAs were determined to be critical to the immune landscape and tumor phenotype of LUSC, based on previous analyses. These critical eRNAs were subsequently examined further for genomic alteration and methylation patterns.

In order to evaluate the effects of tobacco smoke on eRNA expression, we analyzed 129 smoking-onset LUSC samples, 18 nonsmoking-onset LUSC samples, and 49 adjacent normal LUSC samples from TCGA. To eliminate any confounding variables, we ensured that the smoking-onset LUSC samples only consisted of current smokers and excluded former smokers. We also ensured that the nonsmoking-onset LUSC samples only consisted of lifelong nonsmokers. Adjacent normal LUSC samples were used as our control group. We excluded former smokers from both the smoking-onset and nonsmoking-onset LUSC samples because former smokers have a decreased risk of lung cancer than current smokers [13], and smoke cessation may also be associated with decreased somatic mutations and altered molecular profiles. Using differential expression analysis (log fold change (FC) > 1, *p*-value < 0.05), we compared eRNA expression in the smoking-onset LUSC cohort and the lifelong nonsmoking-onset LUSC cohort to adjacent normal LUSC samples. We identified 6060 eRNAs significantly associated with smoking-onset LUSC and 6885 eRNAs significantly associated with nonsmoking-onset LUSC. Specifically, we found 684 downregulated eRNAs and 819 upregulated eRNAs in the smoking-onset LUSC cohort. We also observed that there were significant overlaps in eRNA expression between the smoking-onset LUSC cohort and nonsmoking-onset LUSC cohort, with 2174 eRNAs downregulated and 2358 eRNAs upregulated in both cohorts (Figure 1).

### 3.2. Upregulation of eRNA Expression in Smoking-Onset LUSC Associated with Poor Survival Rates

We analyzed correlations between significantly dysregulated eRNAs and patient survival outcomes. Our results appear to be consistent with existing studies that have suggested that the upregulation of certain eRNAs may be correlated with carcinogenesis, as eRNAs have the potential to be involved in various cancer signaling pathways [9]. Similarly, we have found eRNA expression to be significantly correlated with poor survival, cancer metastasis, and a range of clinical variables.

**Figure 2:**
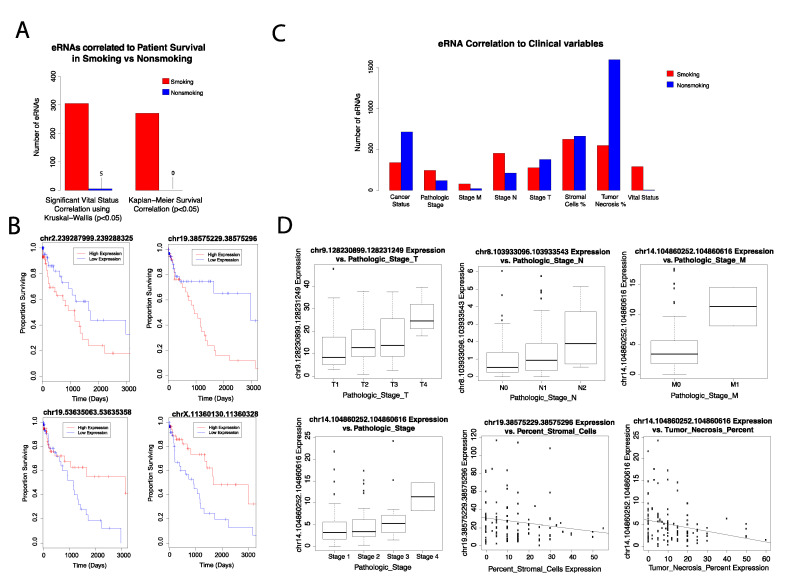
Correlation to Clinical Variables and Patient Survival. (**A**) The total amount of eRNAs identified to be correlated to patient survival and vital status in smoking-onset LUSC and nonsmoking-onset LUSC. Red correlates to smoking-onset LUSC data, and blue correlates to nonsmoking-onset LUSC data. Overall, there was a significantly greater amount of eRNAs associated with patient survival and vital status in the smoking-onset LUSC cohort. (**B**) Select cox proportional hazards regression plots (Kaplan–Meier) of patient survival rates (in days) in correlation to eRNA expression. (**C**) Bar graph representing the total significant correlations between eRNA expression and several clinical variables in both the smoking-onset LUSC cohorts and the nonsmoking-onset LUSC cohorts. Clinical variables investigated include tumor necrosis, cancer status, cancer pathological stage, cancer pathological m stage, cancer pathological n stage, cancer pathological t stage, stromal cell expression, and cancer vital status. (**D**) Select boxplots and scatterplots of eRNAs significantly correlated to clinical variables, including pathological stage T, pathological stage n, pathological stage m, tumor stromal cells, tumor necrosis, and tumor pathological stages 1, 2, 3, and 4. Boxplots plots were created using the Kruskal–Wallis test, while the scatterplots were created using the Spearman test, and all plots were filtered by *p*-value (*p*-value < 0.05). The various correlations in the plots suggest that the upregulation of eRNAs identified from previous analyses are associated with advanced cancer pathological stage and are negatively correlated with tumor stromal cells and tumor necrosis percent expression.

Using the Kaplan–Meier survival analysis, we correlated eRNA expression to patient survival rates in each cohort and plotted the results using Cox proportional hazards regression. We identified 271 eRNAs that were significantly correlated to patient survival in smoking-onset LUSC. Specifically, we found the upregulation of certain eRNAs to be significantly associated with poor patient survival rates (Supplementary Figure S1). However, the downregulation of other eRNAs was correlated with poor patient survival as well (*p*-value < 0.05) (Figure 2). Correlation of eRNA expression to vital status through the Kruskal–Wallis test yielded similar results, with 305 eRNAs significantly correlated to smoking-onset LUSC (Figure 2). Past findings have indicated that various eRNAs are reflective of survival outcomes in cancer patients. For example, CELF2-associated eRNA targets tumor-suppressor gene CELF2, which is expressed in advanced stage stomach adenocarcinoma (STAD) [9]. Therefore, our data provide potential biomarkers for worsened clinical outcomes in LUSC.

### 3.3. Clinical Significance of Smoking Modulated eRNAs on LUSC Pathogenesis and Tumor Development

LUSC is considered a highly heterogeneous cancer, with distinct potentials for tumor phenotype and metastasis [6]. As such, we analyzed the correlations between significantly dysregulated eRNAs and various clinical characteristics in both smoking-onset LUSC and nonsmoking-onset LUSC. More specifically, we correlated eRNA expression to eight distinct LUSC clinical features: tumor necrosis, cancer status, cancer pathological stage, cancer pathological m stage, cancer pathological n stage, cancer pathological t stage, stromal cell expression, and cancer vital status.

Using the Spearman test (*p*-value < 0.05), we found that the upregulation of five eRNAs is associated with decreased tumor necrosis expression in the smoking-onset LUSC cohort. Our analysis with the Kruskal–Wallis test additionally revealed that the increased expression of 11 eRNAs was significantly (*p*-value < 0.05) correlated with tumor status. Conversely, the increased expression of other eRNAs was found to be associated with tumor-free status, indicating that these key eRNAs may be involved in tumor suppressive mechanisms (Figure 2, Supplementary Figure S2).

Lung cancer metastasis is closely related to poor prognosis and survival outcomes in patients and is largely a result of alterations in gene expression, structure, and function [28]. As such, we investigated the correlations between significantly dysregulated eRNAs and cancer metastasis. Specifically, we observed that an increased expression of five distinct eRNAs corresponded to advanced cancer stages. Furthermore, we identified several eRNAs to be associated with metastatic variables. In fact, increased expression of three eRNAs was correlated to advanced cancer pathological m stage (*p*-value < 0.05). Other eRNAs displayed increased expression with cancer pathological stages n and t, demonstrating their potential role in mediating cancer metastasis and proliferation (*p*-value < 0.05).

Our results also indicated that two eRNAs are negatively correlated with increased expression of tumor stromal cells (*p*-value < 0.05) (Figure 2). Stromal cells promote tumor growth by releasing cytokines and growth factors, and the receptors for these ligands are commonly found in cancer cells [29,30]. Advanced-stage tumors often undergo a multitude of interactions with tumor stromal cells and immune cells. Past studies have also indicated that there is a higher number of stromal cells and immune cells corresponding to the expression of eRNA AC003092.1_H in glioblastoma multiforme [31]. Therefore, we predict that the downregulation of these eRNAs may decrease the presence and activity of stromal cells, which could be a possible mechanism for the tumor-suppressive functions of eRNAs.

We then compared the expression of all significant eRNAs in the smoking-onset LUSC cohort and the nonsmoking-onset LUSC cohort. We found that there was an elevated expression of smoking-onset LUSC eRNAs that were directly correlated to advanced cancer pathological stage and cancer vital status, suggesting that tobacco smoke may play a role in mediating eRNA interactions associated with cancer pathogenesis. We also observed that eRNA correlations decreased with advanced cancer TNM staging in the nonsmoking-onset LUSC cohort. As such, we predict that these eRNAs might have a less significant role in mediating worsened LUSC outcomes in nonsmokers. This corresponds to the decreasing trends we observed when nonsmoking-mediated eRNAs are correlated to patient survival and vital status (Figure 2).

However, we found that the number of nonsmoking LUSC eRNAs associated with tumor necrosis percent was significantly higher than that of the smoking-onset LUSC cohort. Tumor necrosis, or necrotic cell death, occurs in advanced tumors, and is associated with poor prognosis [32]. Accordingly, tumor necrosis factor is an inflammatory cytokine that can induce cells to undergo necrotic cell death when apoptosis is blocked [32,33]. Studies have also found that the tumor necrosis factor is significantly higher in smokers, highlighting tobacco’s key role in influencing tumor status [34]. The tumor necrosis factor can act as a tumor promoter, and it is highly involved in various carcinogenic functions, including metastasis and angiogenesis. The tumor necrosis factor may additionally produce reactive oxygen species, which can cause significant DNA damage [3]. Therefore, as the tumor necrosis percent may be representative of tumor necrosis factor, we can expect that the presence of tobacco smoke corresponds to elevated tumor necrosis factor expression in the smoking-onset LUSC cohort. However, as tumor necrosis also induces DNA alterations, we believe that there may be an increased number of mutations in the enhancer regions of DNA, leading to decreased eRNA expression, which is in accordance with our findings.

### 3.4. Smoking-Modulated eRNA Expression Associated with Increased Immune Cell Abundance

Previous studies have established that tobacco smoke significantly impacts the intra-tumor environment of LUSC, often leading to the suppression of NK cell number and cytotoxic T cell activity [15]. However, the mechanisms by which tobacco smoke alters immune cell populations remains relatively unclear. Potential mechanisms that have been studied include tobacco smoke interacting with long non-coding RNA and mRNA, resulting in alterations in immune cell abundance. However, the effects of tobacco smoke on eRNAs, as a mechanism to alter immune cell populations in cancer, remain relatively uncharacterized. Therefore, we assessed the correlations between significantly dysregulated eRNAs and immune cell infiltration using the TIMER software. Analyses were conducted using both the nonsmoking-onset LUSC and smoking-onset LUSC cohorts.

**Figure 3:**
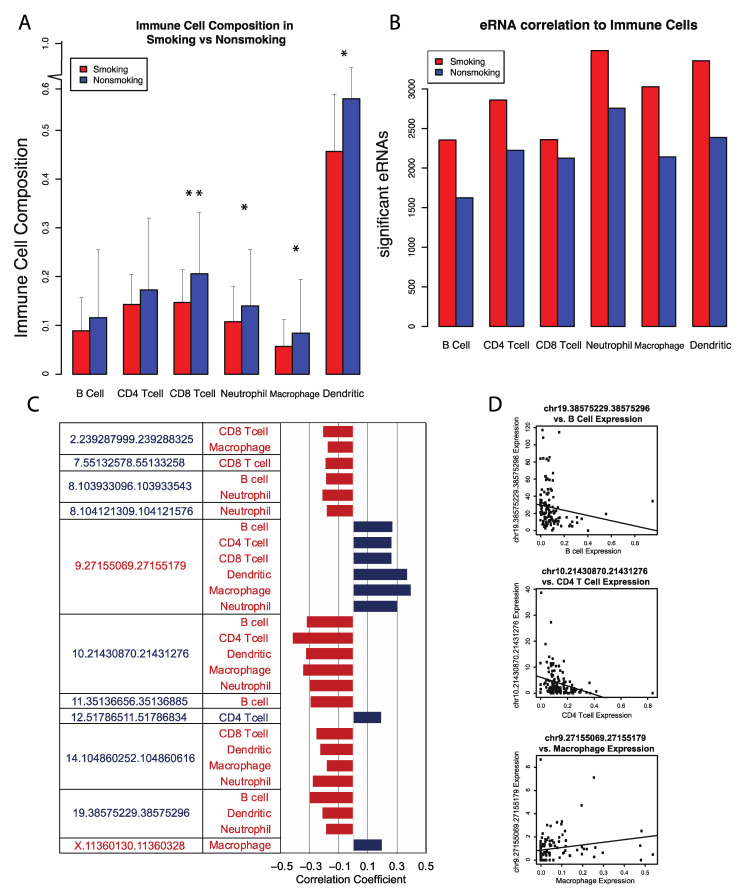
Correlation to immune-associated elements. (**A**) Bar graph comparing immune cell composition in smoking-onset LUSC and nonsmoking-onset LUSC. Red indicates smoking-onset LUSC samples, and blue refers to nonsmoking-onset LUSC samples. Overall, immune cell abundance was higher in nonsmoking LUSC samples. Error bars indicate the standard deviation. Homoscedastic *t*-tests were performed to compare immune cell compositions of the cohorts (** *p* < 0.001, * *p* < 0.05). (**B**) Bar graph comparing significant eRNA correlations to immune cell abundance in smoking-onset LUSC and nonsmoking-onset LUSC. Red indicates smoking-onset LUSC samples, and blue refers to nonsmoking-onset LUSC samples. The data suggest that there is a greater amount of significant eRNAs associated with immune cells in smoking-onset LUSC samples. (**C**) Bar graph summarizing immune cell correlations to the 16 critical eRNAs previously identified to be implicated in the LUSC tumor phenotype and immune environment. Blue indicates relative upregulation, while red refers to downregulation in both eRNA expression and abundance of immune cells. (**D**) Selected scatter plots illustrating the correlations between expression of key eRNAs and immune cell abundance. eRNAs chr19.38575229.38575296 and chr10.21430870.21431276 were downregulated with increasing CD4 and B cell expression, while chr9.27155069.27155179 was upregulated with increasing macrophage expression. The positive and negative correlations between these eRNAs and immune cell abundances suggest potential roles and interactions they might have in promoting LUSC pathogenesis and proliferation.

Overall, immune cell abundance in smoking-onset LUSC samples was lower than that of nonsmoking-onset LUSC samples. Specifically, we observed a decrease in the abundance of dendritic cells, macrophages, CD8 T-cells, CD4 T cells, neutrophils, and B cells. However, despite the decrease in abundance of immune cells overall, we identified a greater number of eRNAs significantly associated with immune cells in the smoking-onset LUSC cohort (Figure 3). As such, we can infer that tobacco smoke may significantly alter eRNA interactions with immune cells and thus decrease immune cell abundance overall, potentially increasing susceptibility to worsened cancer phenotypes.

### 3.5. Identification of 16 Key eRNAs Contributing to Tumor Phenotype and LUSC Immune Landscape

**Figure 4:**
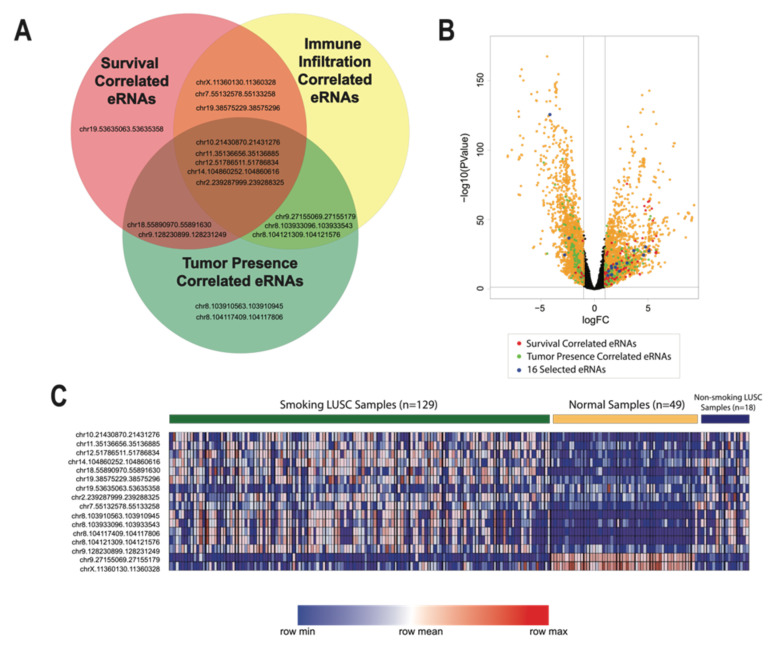
Identification and Analysis of 16 eRNAs identified to be critical to LUSC immune landscape and tumor phenotype. (**A**) Venn diagram summarizing the significant correlations of the selected 16 key eRNAs to patient survival, immune cell infiltration, and tumor status. Each of the 16 selected eRNAs has a high degree of association with various clinical and molecular measures of tumor severity, implicating their potential role in furthering LUSC pathogenesis and metastasis. (**B**) Volcano plot across all significantly dysregulated eRNAs illustrating the statistical significance versus the magnitude of change (log fold change) in survival-correlated eRNAs, tumor-presence-correlated eRNAs, and the identified 16 critical eRNAs. The 16 key eRNAs demonstrate both a high degree of statistical significance and magnitude of change in expression. (**C**) Associations between genomic alterations and eRNA expression in smoking-onset LUSC samples, nonsmoking-onset LUSC, and adjacent normal LUSC samples. Correlations were determined using the REVEALER algorithm (*p*-value < 0.05 and CIC > |0.3|).

We identified 16 key eRNAs that were deemed the most critical contributors to and predictors of advanced tumor phenotype and altered LUSC immune environment. We identified these eRNAs based on correlations to eight clinical and functional variables that are implicated in cancer pathogenesis from past analyses: differential expression, patient survival, cancer pathological stage, pathological m stage, pathological n stage, pathological t stage, stromal cell abundance, and tumor necrosis. All selected eRNAs had a log fold change expression greater than 1, correlations to reduced patient survival, and significant correlations to variables associated with advanced cancer stage and tumor metastasis (*p*-value < 0.05) (Figure 1 and Figure 4). Moreover, we observed that 2 of the 16 identified eRNAs, chr19.53635063.53635358 and chrX.11360130.11360328, were associated with tumor-suppressing genes.

To further examine the roles that these critical eRNAs might have in oncogenesis, we determined the locations of these eRNAs in relation to genes that are deemed critical to LUSC progression. We found that eRNAs chr12.51786511.51786834 and chr7.55132578.55133258 were located in close proximity to the oncogenes GALNT6 [35] and EGFR [36], which promote metastasis and invasion of lung cancer cells. Conversely, eRNAs chr19.53635063.53635358 and chrX.11360130.11360328 were found to be located near the tumor-suppressor genes ZNF415 [37] and ARHGAP6 [38]. Therefore, the interactions of these eRNAs may be influenced by their proximity to oncogenes or tumor-suppressor genes.

We also closely examined immune cell correlations with these 16 key eRNAs. Using the Kruskal–Wallis test, we observed that immune cell abundance was generally negatively correlated with these 16 eRNAs. Specifically, we found that an increase in CD4 and B cell abundance was correlated to the decreased expression of eRNAs chr19.38575229.38575296 and chr10.21430870.21431276, suggesting that reduced interactions between these distinct eRNAs and immune cells may result in immune cell modulation in LUSC (Figure 3). B cells have the potential to inhibit tumor development by producing tumor-reactive antibodies [39]. However, B cells may also promote tumor development through the secretion of autoantibodies and tumor growth factors. Similarly, CD4 cells can cause changes in histone modification and eRNA transcription corresponding to the expression of distinct genes that may or may not have oncogenic properties [40].

Curiously, we found that an increase in macrophage expression was associated with the upregulation of eRNA chr9.27155069.27155179 (Figure 3). Past studies have found that the increased presence of tumor-associated macrophages (TAMs) may result in tumor growth [41]. TAMs can also secrete cytokines and chemokines, such as IL-6 and IL-8, which play significant roles in carcinogenesis, tumor malignancy, and tumor invasion. Therefore, chr9.27155069.27155179 may increase macrophage abundance and further promote malignancy in LUSC.

However, we also observed an increase in macrophage expression with an upregulation of eRNA chrX.11360130.11360328, an eRNA we previously identified to be associated with tumor-suppressive properties (Figure 3). As such, this particular eRNA might be regulating alveolar macrophages, as opposed to TAMs, to potentially promote tumor suppression. Alveolar macrophages are known to possess several antitumor properties, such as cytostasis [42].

### 3.6. Key eRNAs Correlated to Copy Number Variants and Mutations in Smoking-Onset LUSC

**Figure 5:**
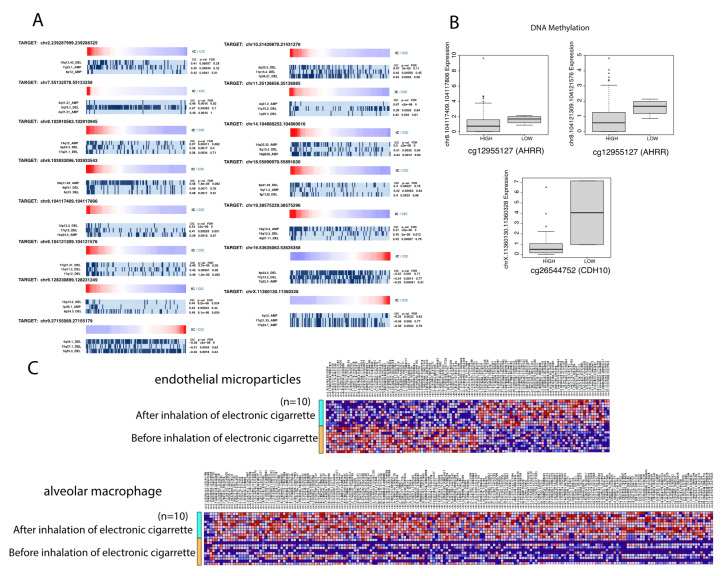
Genomic alteration correlation to 16 key eRNAs via REVEALER (**A**) Mutation heatmaps with red on the left side indicating that an eRNA is overabundant, while red on the left side indicates that the eRNA is under abundant. The following rows below the heatmap specify copy number variant (CNV) type/genetic variants (alteration, deletion, mutation) across all samples. Each bar represents the presence or absence of a CNV in one sample. Correlations were measured using the Repeated Evaluation of Variables Conditional Entropy and Redundancy (REVEALER) algorithm (CIC > |0.3|, *p* < 0.05); 15/16 plots are shown here as the algorithm failed to produce a plot for chr12.51786511.51786834. (**B**) Boxplots correlating eRNA expression with the extent of methylation at CpG sites. In this analysis, the M-values were converted to a binary and categorical variable of “HIGH” and “LOW” in order to conduct the Kruskal–Wallis statistical test. The median abundance level for each eRNA across all samples was calculated. Values lower than the median value were assigned an abundance level of “LOW,” and values above the median value were assigned an abundance level of “HIGH.” The *x*-axis refers to the extent of methylation at the CpG site, and the *y*-axis refers to the eRNA expression level. (**C**) Heatmaps comparing significantly differentially expressed eRNAs in both endothelial microparticles and alveolar macrophages before/after electronic cigarette inhalation (*n* = 10).

It is well established that tobacco smoke causes genetic and molecular damage, as well as increases somatic mutation loads [13,14]. Therefore, it is probable that tobacco smoke might cause chromosomal alterations in enhancer regions of DNA, leading to mutations in eRNA transcripts. As such, we used the REVEALER algorithm to correlate eRNA expression to CNVs and mutation rates. Analyses were conducted using the 16 key eRNAs identified to be critical to the tumor phenotype and immune landscape of LUSC. We found that eRNA expression was strongly associated with a high number of chromosomal alterations and mutations. Specifically, we identified a high number of chromosomal deletions and amplifications. Our results suggest that the presence of tobacco smoke may lead to CNVs and other mutations in the identified eRNAs (as observed in our previous results) (Figure 5). Subsequently, these mutations may play a role in changing the structure and function of these eRNAs. Altered eRNA expression and function might then be correlated to adverse immune cell interactions. We believe this is a possible novel mechanism by which tobacco smoke might affect the molecular landscape of LUSC, contributing to LUSC pathogenesis. However, further studies are required to fully understand the various components of these interactions.

### 3.7. Key eRNAs Correlated to Methylated Sites in Smoking-Onset LUSC

Previous studies have demonstrated that tobacco smoke is associated with altered DNA methylation, particularly in lung cancer [42]. Therefore, we examined the most differentially methylated CpG sites at promoter regions on chromosomes, as well as correlations between these differentially methylated sites and eRNA expression, in smoking-onset LUSC samples and nonsmoking-onset LUSC samples [43]. We found that there were 91,507 differentially methylated CpG sites in smoking-onset LUSC, with 59,005 of these sites having a decreased extent of methylation (*p* < 0.05, log fold change (FC) > 0.1). Curiously, we observed that only 50,198 sites, out of 94,519 differentially methylated CpG sites, had a decreased extent of methylation in the nonsmoking-onset LUSC cohort (*p* < 0.05, log fold change (FC) > 0.1). Thus, it appears that there is a greater amount of hypomethylated sites in the smoking-onset LUSC cohort, suggesting that tobacco smoke may lead to decreased methylation patterns.

In order to more closely examine the effects of methylation on eRNA expression, we assessed patterns of differentially methylated CpG islands associated with the expression of the 16 key eRNAs we previously identified as critical to the immune landscape and tumor phenotype of LUSC. The most commonly methylated sites occurred at chromosomes 1, 2, and 7 and were associated with the expression of eRNAs chr8.104117409.104117806, chr8.104121309.104121576, and chrX.11360130.11360328, respectively. For many of these correlations, a low extent of methylation at these sites corresponded to the expression of these specific eRNAs. As our previous results indicate that chr8.104117409.104117806 and chr8.104121309.104121576 were also found to be upregulated with advanced tumor status and pathological stage, our data suggest that tobacco smoke leads to the hypomethylation of these particular sites, resulting in the increased expression of eRNAs associated with worsened LUSC outcomes.

Furthermore, past studies have found that tobacco smoke often leads to the hypomethylation of critical genes, such as AHRR—which is highly implicated in lung cancer risk [44,45]. In fact, studies have found that smokers had 19% lower methylation at the AHRR CpG site than nonsmokers. Our findings are consistent with existing literature, as we found that there was hypomethylation at several AHRR sites in smoking-onset LUSC. We additionally observed that the CDH10 site, which has been established as a tumor suppressor in lung cancer, was correlated with the increased presence of eRNA chrX. 11360130.11360328 [46]. We have previously found that this eRNA has various tumor-suppressive properties as well.

### 3.8. Identification of eRNAs Associated with Electronic Cigarette Smoke

To evaluate the role of e-cigarette smoke on differential eRNA expression [27], we analyzed mRNA-sequencing datasets that examined the effect of acute electronic cigarette smoke inhalation on 10 lifelong nonsmoking patients. The datasets quantified the effect of electronic cigarette smoke on the lungs using sequenced mRNA data from endothelial microparticles and alveolar macrophages. To eliminate confounding variables, we aimed to identify the effect of electronic cigarette smoke on lung eRNA expression using two separate cohorts. In the first cohort, we compared the differential expression of lung eRNAs in lifelong nonsmokers before and after electronic cigarette use, with endothelial microparticles as the primary measure of lung alteration. In the second cohort, we compared the differential expression of lung eRNAs in lifelong nonsmokers before and after electronic cigarette use with respect to alveolar macrophage transcriptome alterations.

Using differential expression analysis, we identified 194 significantly dysregulated lung eRNAs in the alveolar macrophage transcriptome quantified cohort and 108 significantly dysregulated eRNAs in the endothelial microparticle quantified cohort (*p* < 0.05, log fold change (FC) > 1). We additionally observed a large upregulation of eRNAs associated with electronic cigarette use in the alveolar macrophage cohort (Figure 5).

Interestingly, we observed that several of the 16 key eRNAs we previously identified to be critical to the LUSC immune landscape and tumor phenotype were also present following electronic cigarette use in the alveolar macrophage cohort. Specifically, we found that 10 key eRNAs were present in the alveolar macrophage transcriptome cohort, and 9 of these eRNAs were upregulated in the presence of electronic cigarette smoke. While these eRNAs did not meet our threshold of a log fold change value greater than 0.1, we found that six of these eRNAs had log fold change values above 0.6. Therefore, our results suggest that electronic cigarette smoke may increase the expression of key eRNAs that are also associated with tobacco-smoke-onset LUSC, thus potentially acting through similar molecular mechanisms as tobacco-smoke-mediated LUSC eRNAs to promote worsened clinical outcomes.

Accordingly, previous studies have found that e-cigarette devices contain harmful substances that are often considered carcinogenic [16,47]. For example, e-cigarette devices have various carbonyl compounds, such as acetaldehyde and formaldehyde, which have inflammatory and toxic effects. The inflammation induced by these particular substituents has been found to lead to the activation of macrophages and the generation of reactive oxidative species, which can result in greater immune modulation and induce the production of inverse inflammasomes [18]. As such, an immunosuppressive environment is created, with an increased hostility towards T cells, which is also commonly associated with lung cancer oncogenesis and malignancy.

However, e-cigarettes also contain substituents that act through alternative mechanisms to cause cancer. Cancer is a genetic disease and is associated with genetic damage, defects, and alterations. These genetic alterations may be caused by etiological agents that have the potential to cause significant DNA damage [17]. Accordingly, e-cigarettes contain a myriad of substances that are capable of creating DNA breakages. These toxic compounds cause DNA repair malfunction, cell cycle malfunction, mitochondrial dysfunction, and the formation of reactive oxygen species, thus leading to malignancy and oncogenesis. Moreover, 10% of the nicotine substances in electronic cigarette devices undergo a conversion to nitrosamine, increasing the risk for nitrosamine carcinogenesis. Previous studies have found that nitrosamine carcinogenesis is caused by increased DNA methylation and agonistic activity on the nicotinic acetylcholine receptor, which promotes tumor growth [18]. E-cigarettes have also been found to contain acrolein, a substance that is commonly associated with lung damage, chronic obstructive pulmonary disease (COPD), and lung cancer. Acrolein directly creates adducts with DNA, often binding at cytosine-methylated CpG sites or near tumor-suppressor mutational spots, resulting in increased DNA damage as well as reducing the effectiveness of DNA repair mechanisms [48].

In accordance with past findings, we predict that as a result of harmful substituents, e-cigarette smoke will cause DNA breakages and mutations. Therefore, it is likely that the enhancer regions of DNA will also have various alterations, leading to changes in eRNA structure, function, and expression. Thus, eRNAs with adverse functions might be upregulated, as observed in our data, directly promoting LUSC tumor phenotypes.

Previous studies have also found that e-cigarette smoke may suppress immune cell abundance in the lungs. For example, macrophages were found to have impaired function following e-cigarette smoke exposure, with effects similar to that of tobacco smoke or COPD [49]. Alveolar macrophages play a critical role in removing allergens or other small particles and have also been found to secrete proinflammatory cytokines that are implicated in several antitumor functions [42].

Therefore, similar to our findings regarding the association between tobacco-smoke-mediated LUSC eRNAs and immune cell abundance, we predict that e-cigarette-smoke-mediated upregulation of eRNA expression may lead to an increased amount of adverse eRNA interactions with immune cells, thus decreasing their abundance and promoting LUSC progression. However, further experimentation is required to confirm this novel conclusion.

## 4. Discussion

To the best of our knowledge, we are the first to profile the effects of tobacco smoke and e-cigarette smoke on eRNA expression in LUSC pathogenesis. Previous studies examining eRNA expression in LUSC suggest that eRNAs are highly implicated in decreased survival rates and poor clinical outcomes [9,10,11]. Previous findings also indicate that LUSC is significantly influenced by etiological agents [9,12,15]. Furthermore, studies have found that tobacco-smoke-onset LUSC has distinct eRNA expression, thus indicating that tobacco smoke might play a role in regulating eRNA expression to promote worsened clinical outcomes.

Using RNA-sequencing TCGA data from 129 smoking-onset LUSC samples, 18 nonsmoking-onset LUSC samples, and 49 adjacent normal LUSC samples, we identified 684 downregulated eRNAs and 819 upregulated eRNAs associated with smoking-onset LUSC. Next, we analyzed these differentially expressed eRNAs in respect to LUSC pathogenesis by examining correlations to several clinical variables. Specifically, we observed that eRNAs in smoking-onset LUSC were closely associated with eight LUSC clinical features pertaining to tumor phenotype and metastasis: tumor necrosis, cancer status, cancer pathological stage, cancer pathological m stage, cancer pathological n stage, cancer pathological t stage, stromal cell expression, and cancer vital status.

In addition to various clinical correlations, we aimed to assess how tobacco smoke might also influence immune cell abundance. Studies have found that tobacco smoke can suppress immune cell abundance and may modulate immune cell long non-coding RNA interactions to promote metastasis [15].

However, tobacco-smoke-mediated eRNA–immune cell interactions in LUSC have not been extensively investigated. As such, we correlated immune cell abundance to eRNA expression in both smoking-onset LUSC and nonsmoking-onset LUSC. We observed an overall decrease in immune cell abundance in smoking-onset LUSC in comparison to nonsmoking-onset LUSC. However, despite the decrease in immune cell abundance overall, we found that there was an increase in significant eRNAs associated with smoking-onset LUSC immune cells. Our findings are consistent with previous studies that have indicated that tobacco smoke may suppress immune cell abundance^15^. Immune cells are critical defense mechanisms against cancer, with various immune cells holding the capability to directly kill tumor cells [50]. T follicular cells are correlated with better patient survival, while CD4 cells can facilitate the proliferation of B cells to better target tumor cells [51]. Thus, by decreasing the abundance of immune cells, tobacco smoke directly weakens immune defense mechanisms against cancer and reduces favorable prognosis. Our results also suggest that a distinct cohort of eRNAs are closely related to immune cell abundance in smoking-onset LUSC, indicating that tobacco smoke may lead to adverse eRNA interactions with immune cells, reducing their abundance.

Next, we characterized the key eRNAs that are predictive of and contribute to advanced tumor phenotype and the altered LUSC immune landscape. We selected 16 key eRNAs based on several variables from previous analyses that suggest significant implications in cancer proliferation and pathogenesis.

We found that several of these 16 key eRNAs in the smoking-onset LUSC cohort were in close proximity to oncogenes and tumor-suppressor genes. For example, we observed that eRNAs chr12.51786511.51786834 and chr7.55132578.55133258 were upregulated and located near GALNT6 [35] and EGFR [36], which have been found to promote metastasis of lung cancer cells. Accordingly, previous studies have shown that the activation of oncogenes can promote enhancer activity and the production of eRNAs with oncogenic properties [9]. Similarly, we found that eRNAs chr19.53635063.53635358 and chrX.11360130.11360328 were downregulated and located near ZNF415 [37] and ARHGAP6 [38], which have been identified as tumor-suppressor genes. It has been established that tumor-suppressor genes face a loss of function in human cancer [52]. Therefore, our results demonstrate that tobacco smoke might silence key tumor-suppressor genes, thus also silencing nearby enhancers and subsequently leading to the downregulation of eRNAs with tumor-suppressive properties [8].

Next, we examined the effect of tobacco smoke on methylation to identify potential mechanisms by which eRNA expression may be affected by the presence of etiological agents. We examined correlations between methylated CpG sites and the expression of eRNAs implicated in LUSC pathogenesis. We found that hypomethylated CpG regions in our tobacco-smoke onset LUSC cohort were significantly associated with eRNAs that we have previously determined to promote advanced tumor stage and worsened clinical outcomes. Our results suggest that these specific sites might be methylated in the absence of tobacco smoke and therefore exhibit reduced expression of eRNAs known to exacerbate LUSC risk. In the presence of etiological agents, such as tobacco smoke, our findings indicate that there is increased hypomethylation and, therefore, an upregulation of eRNAs corresponding to LUSC risk, metastasis, and pathogenesis. However, we also observed a lower extent of methylation corresponding to eRNA chrX.11360130.11360328, which we previously identified to be associated with tumor-suppressive properties. Recently, multicancer detection tests that utilize methylation patterns for early cancer detection have been developed for clinical use. This particular detection tool analyzes methylation patterns specific to each cancer and common across all cancers in order to distinguish between cancer and normal samples as well as identify cancer signal origin [53]. Thus, the results of our study may provide more accurate representations of methylation patterns in tobacco-smoke-mediated LUSC, as well as increase the diagnostic utility of this particular detection mechanism across a wider range of individuals.

In addition to investigating the impact of tobacco smoke on eRNA expression in LUSC, we also examined the effect of e-cigarette smoke on eRNA expression and immune cell abundance. E-cigarettes are commonly used to aid with smoke cessation and control [18,54]. However, there are concerns that e-cigarette smoke may be implicated in lung cancer [18]. E-cigarettes contain a myriad of carcinogenic substances, such as carbonyl compounds, which have the potential to cause DNA breaks and inhibit DNA repair [18]. As cancer is largely associated with DNA alterations and mutations, these substances directly increase cancer risk and promote advanced tumor phenotypes [17].

Thus, we found that there was a large upregulation of eRNAs associated with e-cigarette smoke inhalation. We observed that several of the 16 key eRNAs that we previously identified to be critical to tobacco onset LUSC were also present following exposure to e-cigarette smoke, suggesting that tobacco smoke and e-cigarette smoke may act through similar mechanisms to influence LUSC outcomes.

As such, we predict that e-cigarette-smoke-dysregulated eRNAs may engage in adverse interactions with immune cells to decrease their abundance, similar to dysregulated eRNAs in tobacco-smoke-onset LUSC. Previous studies have found that e-cigarette smoke impairs macrophage function [50]. Certain macrophages, such as alveolar macrophages, play critical antitumor roles [42]. Thus, impairment in the function of these key macrophages might result in tumor promotion and worsened clinical outcomes. Moreover, studies have also found that there is an increased presence of lipid-laden macrophages correlated to e-cigarette or vaping product use-associated lung injury [55]. Lipid-laden alveolar macrophages are often indicators of aspiration and may serve as markers for lung disease [56,57]. Therefore, e-cigarette smoke may be correlated with an increase in lipid-laden macrophages, thus potentially promoting LUSC disease severity. Accordingly, we propose that e-cigarette smoke modulates immune cell abundance through the expression of these eRNAs.

Overall, our study provides a comprehensive overview of the mechanisms by which tobacco smoke and e-cigarette smoke influence eRNA interactions to promote LUSC pathogenesis, proliferation, and metastasis. Our findings offer potential biomarkers and diagnostic targets for LUSC, as well as provide insight into how various etiological agents might further disease progression on a molecular level. Moreover, our results suggest that investigating the impact of tobacco smoke on various other genetic and transcriptional factors is key to elucidating alternative modes of LUSC pathogenesis.

## 5. Conclusions

In summary, our study provides key insights on specific eRNAs and their influence on smoking-mediated LUSC. We identified and analyzed the effects of tobacco smoke on eRNA expression and the influence of smoking-mediated eRNA dysregulation on LUSC pathogenesis. We identified that while eRNAs in the nonsmoking LUSC cohort did not correlate to patient survival, many dysregulated eRNAs in smoking-onset LUSC were significantly correlated with patient survival, immune cell abundance, and advanced LUSC pathological stage. Of the dysregulated eRNAs in smoking-onset LUSC, we continued to filter and identify 16 eRNAs that we considered the most critical contributors to and predictors of an altered immune landscape and advanced tumor phenotype in LUSC. A total of 14 of the 16 eRNAs were highlighted as oncogenic, as they were correlated with reduced patient survival, positively associated with advanced cancer stage and tumor metastasis, and had a log fold change expression greater than 1. Of the 16 eRNAs, two were highlighted as tumor-suppressive as they were correlated with increased patient survival, negatively associated with advanced cancer stage and tumor metastasis, and had a log fold change expression of less than −1.

We also found that these 16 key eRNAs critical to the LUSC pathogenesis were associated with a high degree of chromosomal alterations and a decreased extent of DNA methylation at key sites. Given these 16 eRNAs’ strong association with clinical outcomes, they could serve as prognostic markers in smoking-onset LUSC. In addition, we found that several of these 16 key eRNAs were dysregulated in the presence of e-cigarette smoke. To the best of our knowledge, we are the first to profile significantly dysregulated eRNAs associated with e-cigarette vapor, providing a novel mechanism by which vaping may contribute to LUSC pathogenesis. Future research using in vitro and in vivo experiments is necessary to validate our current findings on the influence of eRNAs on LUSC progression.

## Data Availability

Publicly available datasets were analyzed in this study. These data can be found here: https://www.ncbi.nlm.nih.gov/geo/query/acc.cgi?acc=GSE85121 (accessed on 22 March 2021) and https://portal.gdc.cancer.gov/projects/TCGA-LUSC (accessed on 12 March 2019).

## Supplementary Materials

The following are available online at https://www.mdpi.com/article/10.3390/cancers13164225/s1, Figure S1: Significant Kaplan–Meier plots of the 16 key eRNAs, Figure S2: Additional various clinical variable box plots of the 16 key eRNAs.
