# Supplementary figures and images for: Tobacco Smoke and Electronic Cigarette Vapor Alter Enhancer RNA Expression That Can Regulate the Pathogenesis of Lung Squamous Cell Carcinoma
Source: Cancers (Basel). 2021 Aug 23;13(16):4225. doi: 10.3390/cancers13164225 (PMC8391195; doi:10.3390/cancers13164225)

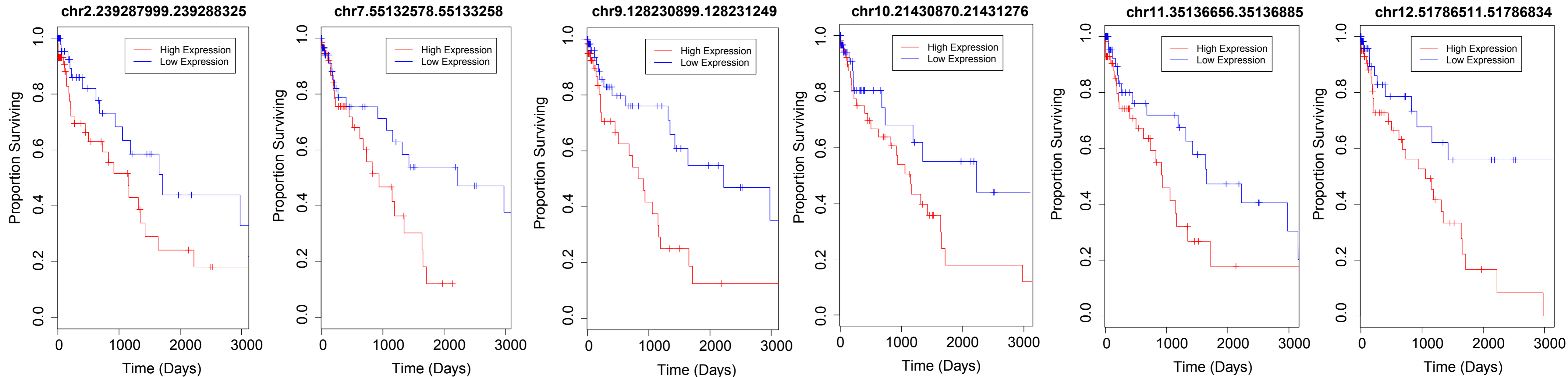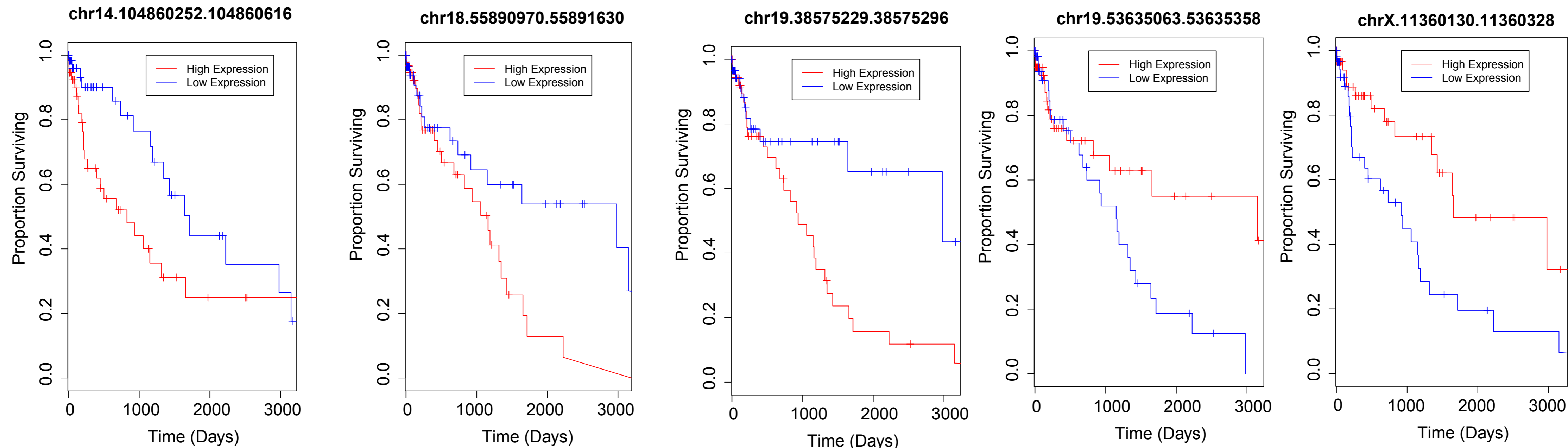

Supplement: Supplementary file 1 [file cancers-13-04225-s001.zip › Figure S1.pdf]

A

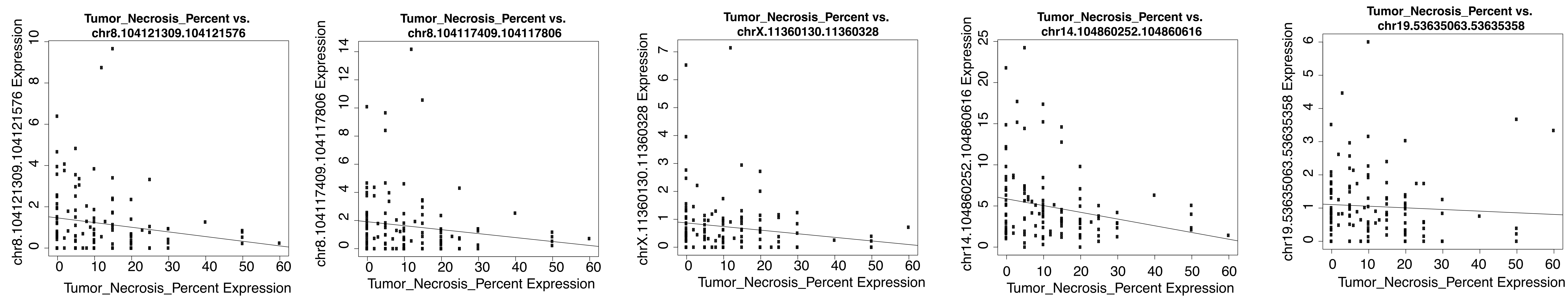

B

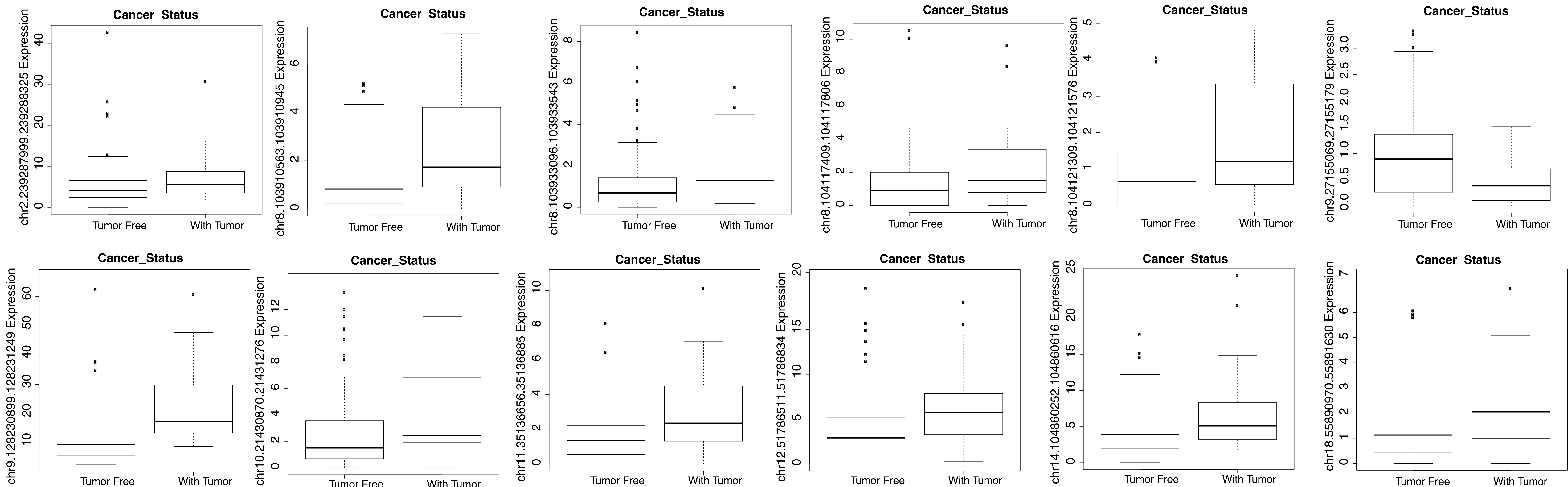

C

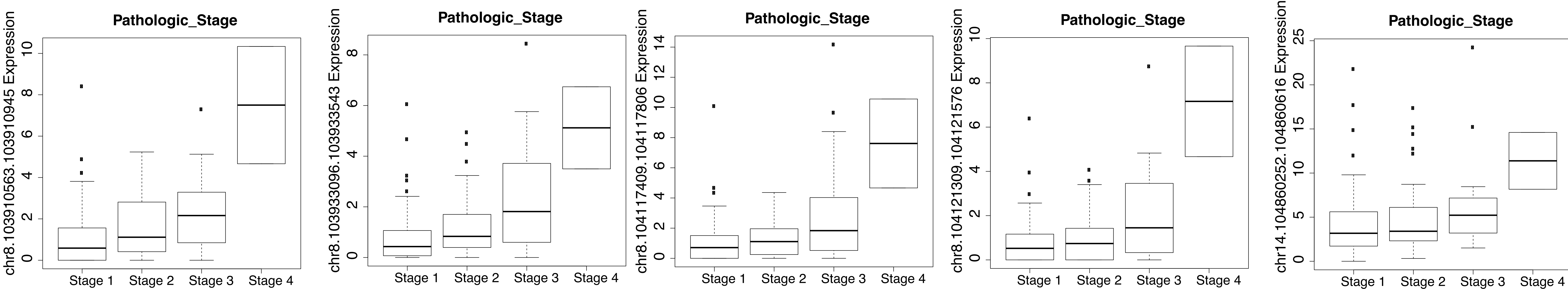

D

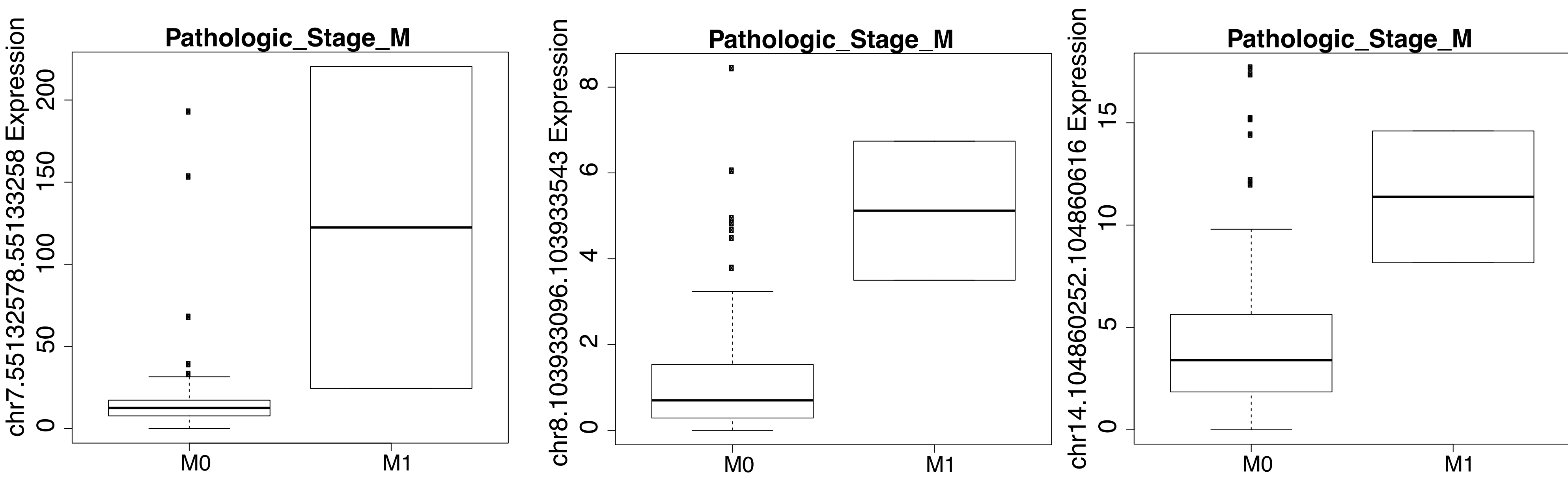

E

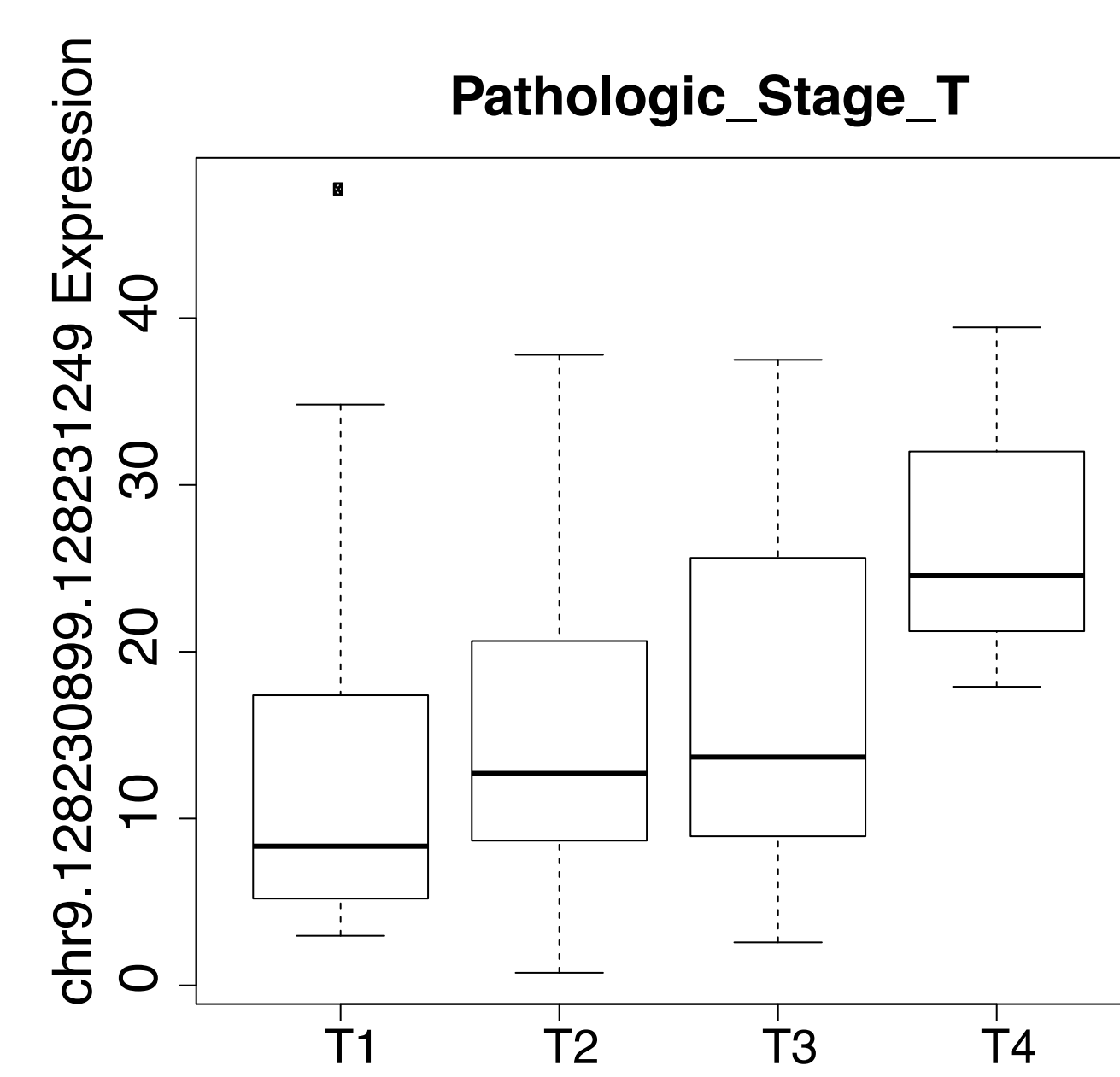

F

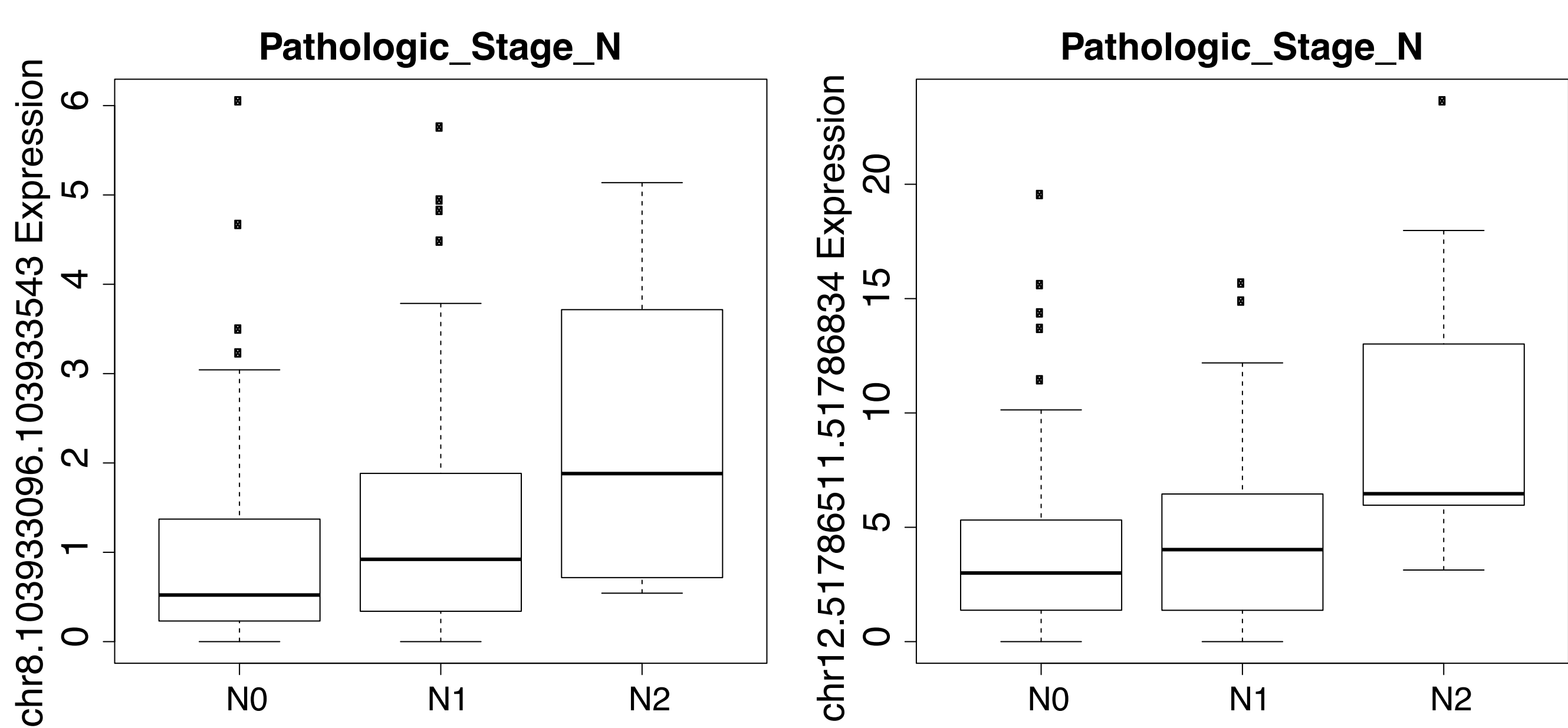

G

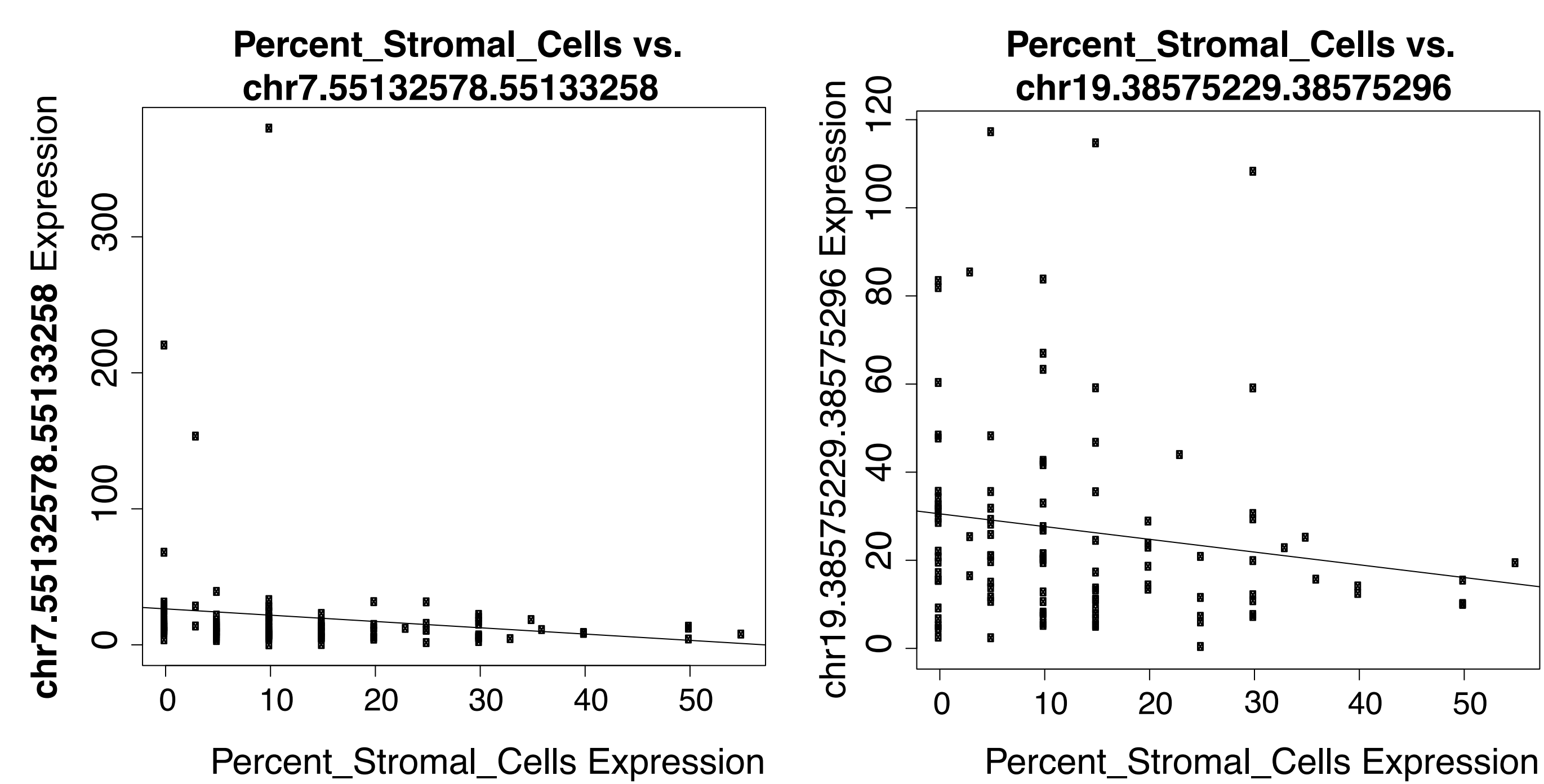

Supplement: Supplementary file 1 [file cancers-13-04225-s001.zip › Figure S2.pdf]
